# Dual Action of *Beauveria bassiana* (Hypocreales; Cordycipitaceae) Endophytic Stains as Biocontrol Agents against Sucking Pests and Plant Growth Biostimulants on Melon and Strawberry Field Plants

**DOI:** 10.3390/microorganisms10112306

**Published:** 2022-11-21

**Authors:** Spiridon Mantzoukas, Eufrosini Daskalaki, Foteini Kitsiou, Vasileios Papantzikos, Dimitrios Servis, Stergios Bitivanos, George Patakioutas, Panagiotis A. Eliopoulos

**Affiliations:** 1 Department of Agriculture, Arta Campus, University of Ioannina, 45100 Ioannina, Greece; 2 Laboratory of Plant Physiology, Department of Biology, University of Patras, 26504 Patras, Greece; 3 BASF Hellas S.A., 15125 Marousi, Greece; 4 Laboratory of Plant Health Management, Department of Agrotechnology, University of Thessaly, Geopolis, 41500 Larissa, Greece

**Keywords:** entomopathogenic fungi, endophytes, biological control, aphids, thrips, biostimulant, plant growth

## Abstract

Entomopathogenic fungi (EPF) can colonize plant tissues and serve crops not only as biopesticides but also as biostimulants that promote plant growth and trigger defense mechanisms. In this context, field trials were conducted evaluating two commercial strains of the entomopathogen *Beauveria bassiana* (Hypocreales: Cordycipitaceae), GHA (Botanigard) and PPRI 5339 (Velifer^®^ ES) and a wild strain (AP0101) isolated from Achaia, Greece. The three strains were investigated in the field for their endophytic effects on melon *Cucumis melo* (Cucurbitales: Cucurbitaceae) and strawberry *Fragaria* sp. (Rosales: Rosaceae) plants and in particular for their ability to colonize plant tissues, control infestations of sucking insects *Aphis gossypii* (Hemiptera: Aphididae), *Chaetosiphon fragaefolii* (Hemiptera: Aphididae) and *Frankliniella occidentalis* (Thysanoptera: Thripidae), and improve plant growth parameters (plant height, number of flowers and fruits). All experimental fungal strains successfully colonized both plants. A significant decrease in the aphid and thrip populations was observed in the treated plants compared to the untreated control. As for plant growth, the number of flowers and fruits was significantly increased in plants treated with *B. bassiana* strains AP0101 and PPRI 5339. Our results clearly indicate that fungal endophytes can efficiently act as dual action agents demonstrating both insecticidal and growth-promoting effects.

## 1. Introduction

One group of insects of great importance to agriculture and the integrated pest management (IPM) program are the sucking pests. The piercing-sucking mouthparts of these pests have evolved to penetrate plant tissue by piercing it and sucking out the juices, causing damage while carrying pathogenic microorganisms and transmitting diseases to the plants [1]. Heavily infested plants show symptoms, such as leaf chlorosis, wilting and malformations and may eventually die [2]. Among the harmful sucking pests, the cotton or melon aphid *Aphis gossypii* (Glover), the strawberry aphid *Chaetosiphon fragaefolii* (Cockerell) (Hemiptera: Aphididae) and the Western flower thrip *Frankliniella occidentalis* (Pergande) (Thysanoptera: Thripidae) are of great interest because in all their developmental stages, they deprive plants of nutrients [2,3,4,5] and at the same time transmit viral diseases [3,4,5,6,7], resulting in significant financial losses in the production of commercially important crop species, such as melons and strawberries.

Among non-chemical pest control methods, biological control by entomopathogenic fungi (EPFs) is one of the most effective, environmentally and human friendly alternatives. EPFs have several advantages over conventional insecticides. Some EPFs have the ability to grow endophytically. Fungal endophytes are quite common in nature, and some of them have extremely unfavorable properties for insects, nematodes and plant pathogens [8]. Endophytes have been identified in several commercially important plant species [8,9,10] and act in various ways as promoters of plant growth [11] or as beneficial rhizosphere colonizers [12]. They can be used as biofertilizers [9] and vertically transmitted fungal endophytes [13]. EPF endophytes promote plant growth, by producing various secondary metabolites, that have a unique species-specific bioactive structure (e.g., benzopyranones, phenolic acids, quinones and steroids) [14] and are widely used for their agrochemical, antibiotic, antiparasitic and antioxidant properties [15]. The endophyte *Beauveria bassiana (Bals.-Criv.) Vuill.* (Hypocreales: Cordycipitaceae), is the best-studied EPF and has a wide range of plant hosts and can be found as a natural endophyte, suggesting that it has complex life cycles that can be completed in plants, soil, or invertebrates [16]. Moreover, recent studies on commercial endophytic strains of *B. bassiana* have highlighted its ability to not only promote plant growth but also provide systemic protection against pests and pathogens [17,18], placing this EPF at the top of the list of promising biological agents to improve crop production.

In the present study, a field trial was conducted testing three strains of the EPF *B. bassiana*: two commercial strains and one wild strain isolated from the first author (S. Mantzoukas). These strains were inoculated on melon and strawberry plants. After application, both crops were monitored to study the effects of EPF inoculation on plant growth and on the population of *A. gossypii* on melon and *F. occidentalis* and *C. fragaefolii* on strawberry plants.

## 2. Materials and Methods

The field experiment was conducted in two different regions of Greece. Experiments on melon plants (*Cucumis melo* var Moonshine) were implemented in a field in Konitsa (Ioannina Prefecture) (Lat: 40.02749°, Long: 20.72416°) from May to September in 2022 and in a strawberry field (*Fragaria* sp. var Fortuna) in Varda (Ilia Prefecture) (Lat: 38.02614°, Long: 21.35375°) from November 2021 to June 2022.

During the field experiments, three *Beauveria bassiana* isolates (two commercial and one wild) were evaluated: strain GHA 10.735% (Botanigard 10,7SC, K&N Efthymiadis Single Member S.A., Thessaloniki, Greece), strain PPRI 5339, contained in the EPF-based biopesticide Velifer^®^ ES (BASF SE, Florham Park, NJ, USA) and a wild strain named AP0101 collected from soil samples of olive orchards in Glafkos (Achaia, W. Greece) [19]. The isolates were maintained in Petri dishes on SDA medium (Sabouraud Dextrose Agar, Oxoid) at a temperature of 5 ± 1 °C and renewed monthly.

### 2.1. Field Experiment Setup

**Figure 1:**
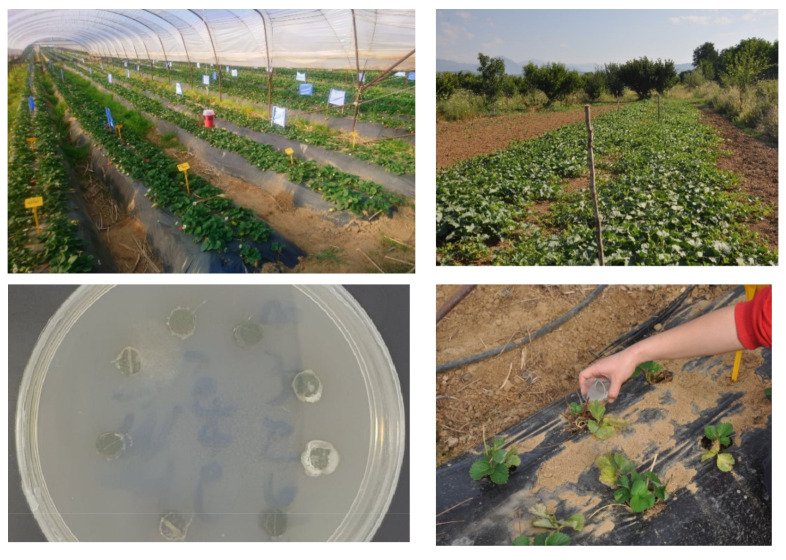
Experimental fields of strawberries (**up left**) and melons (**up right**), leaf pieces from treated plants in Petri dish showing *B. bassiana* hyphae growth (**down left**), watering experimental plant with 10 mL of conidial solution (**down right**).

In both fields, four plots were selected for each treatment, including the control, (2 × 0.8 m strawberry plant plots and 4 × 8 m melon plant plots), each containing twenty plants. For melon, planting was carried out at 0.80 m apart along two lines of each plot, with the lines 1.2 m apart. For *strawberries*, planting was done in raised beds and at a spacing of 0.60 m between rows and 0.20 m between the plants in each row (Figure 1). All recommended agronomic practices (irrigation, fertilization) were applied to both crops. All strawberry and melon plants were watered daily with drip irrigation. Fertilization was applied before transplantation and was identical for all plants (80–100 Kgr/acre of NPK 12-12-17 + 2MgO). No other crop protection treatments took place.

Conidial suspensions of 10^8^ conidia/mL were prepared for the isolates of *B. bassiana* [20]. Conidial viability (%) was calculated by counting a total of 100 conidia per fungal isolate. Fungal isolates with a viability of ≥95% were used for the bioassays. A solution of H_2_O + Tergitol NP9 0.05% was used as control. The application of the three fungal treatments and the control in the experimental field was completely random. As soon as the four-leaf stage was reached, 10 mL of the EPF conidial suspensions were applied (watered) to the roots of the melon and strawberry plants (Figure 1). For the strawberry plants, a second irrigation (identical to the first) with conidial suspensions was carried out in March 2022, as the cultivation takes eight months (twice as long as for the melon).

### 2.2. Assessment of Insect Infestation

During the 2022 growing season, leaf samples were taken from melon plants every 15 days from May to September. A total of 80 plants were sampled using the Cluster sampling method: 4 clusters (plots), 20 plants in each cluster at each sampling point. For each sampling, 25 leaves of the same age and size, were collected from the middle of the plant, with sterile scissors from each plot. The number of 3rd–4th instar nymphs of *A. gossypii* per leaf was counted visually in the field using a ×7 head lens (optiVISOR, LightCraft, London, UK). These developmental stages were preferred over adult and juvenile nymphs for counting as a population indicator, as this is more practical and reliable.

Strawberry plants were sampled in the same way as melon, every 15 days from November 2021 to June 2022. The number of *F. occidentalis* adults per flower was counted visually in the field. The adult thrips were counted rather than those in the immature stages, as this is easier for farmers and visual counting of immature thrips is unreliable [21]. In addition to the thrips, 3rd–4th instar nymphs of *C. fragaefolii* were also counted per leaf on strawberry plants, just as in melon plants.

### 2.3. Assessment of B. bassiana Colonization in Inoculated Plants by Re-Isolation and In Vitro Culturing

To investigate *B. bassiana* colonization, the sampled leaves, were taken to the laboratory after insect counting was completed. There, they were surface sterilized by immersion in a 96% ethanol solution for one minute, then in a 6% sodium hypochlorite solution for five seconds and finally again in a 96% ethanol solution for thirty seconds [20]. Then, eight (8) cylindrical pieces 1 cm long and 0.5 cm thick were cut from each leaf in a laminar air flow (Vertical Air Laminar Flow Cabinet Clean Bench, Equip Mechanical Applications LTD, Piraeus, Greece). Next, the sterile leaf pieces were transferred to Petri dishes containing SDA substrate using a sterile metal hook and incubated in the dark at 25 ± 2 °C and 80% humidity. Germination of fungal conidia (with visible hyphal growth) on the melon or strawberry leaf pieces was assessed after 14 days using an optical microscope (40×) (Figure 1). The number of leaf pieces that displayed fungal growth was recorded.

### 2.4. Effect on Plant Growth

The effect of the tested endophytic strains of *B. bassiana* on the plant growth was also evaluated. Both plant species were monitored throughout the cultivation period for various growth parameters. More specifically, the number of leaves, flowers and fruits. as well as the height of the plants, were counted every fortnight. The height was measured on site with a tape measure.

### 2.5. Statistical Analysis

The growth parameters of the plants (number of leaves, flowers and fruits, plant height), colonization intensity (% of colonized leaf pieces per plant) and pest infestation levels (aphids/leaf, thrips/flower) were subjected to statistical analysis. The differences in growth parameters and pest infestation levels among various treatments were examined using one-way ANOVA (Tukey-Kramer test). The percentages of colonization were arcsine transformed before analysis. A three-way ANOVA was performed to evaluate the main effects and interactions of the three main factors: the plant species, the *B. bassiana* strain and the time interval (days) after treatment. All statistical analyses were carried out using SPSS (SPSS Inc., Armonk, NY, USA, ver. 25) [22].

## 3. Results

### 3.1. Endophytic Control Tests

A key trait for successful establishment of endophytic biocontrol agents is the ability to colonize host plant tissues. Successful colonization of *Cucumis melo* and *Fragaria* sp. leaves by three strains of *B. bassiana* was evaluated seven-, fifteen- and thirty-days after inoculation (treatment). Identification of *B. bassiana*, was done by macroscopic observation and microscopic examination, following the protocol of Barnett and Hunter [23]. The main characteristics for the identification of *B. bassiana* are the white muscardine (mycelium and conidia are white), the single oval-shaped conidia and the zigzag pattern of the conidiophore.

All tested strains of *B. bassiana* were very effective at colonizing plant tissues, achieving a 100% colonization rate (number of plants colonized/number of plants treated). Observations on SDA petri dishes with leaf pieces confirmed that all treated plants were successfully colonized (*B. bassiana* was present in at least one leaf piece of each plant). On the other hand, no EPF was detected in the leaf pieces of the control plants.

**Figure 2:**
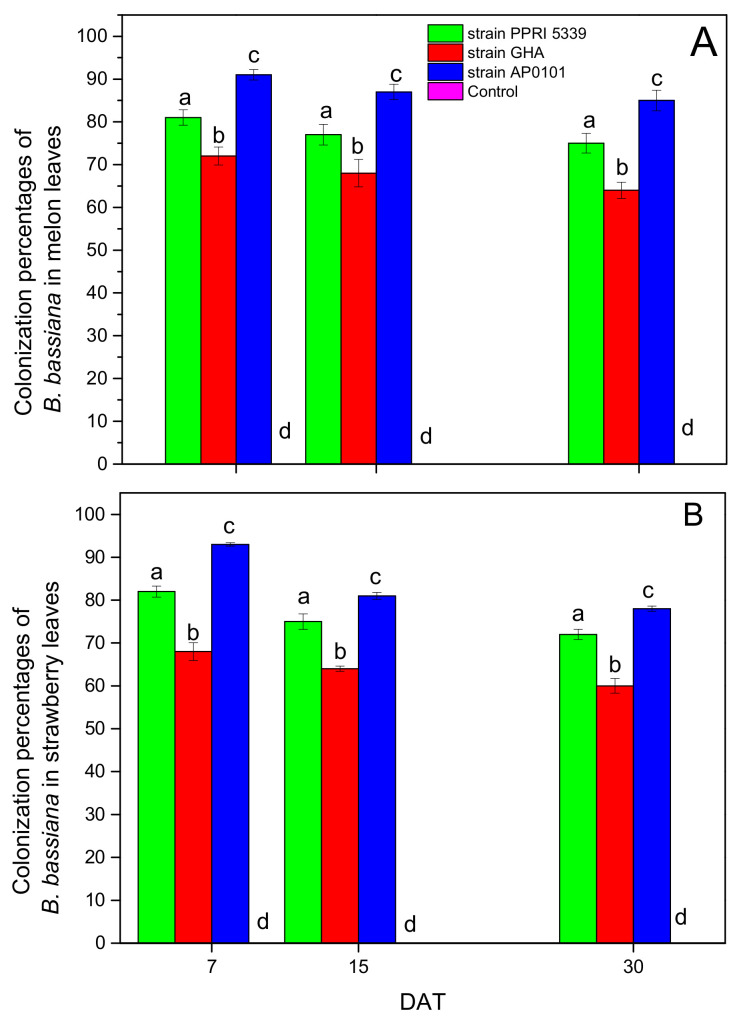
Mean percentage of colonized leaf pieces (% ± sd; *n* = 200 pieces/treatment) in (**A**) *C. melo* leaves (**B**) *Fragaria* sp. leaves. Treated plants were watered with 10 mL of *B. bassiana* conidial suspension of 10^8^ conidia/mL at the stage of four leaves, 25 leaves were sampled from each treatment plot, 8 pieces from each leaf were examined, means of the same plant and DAT marked with the same letter did not differ significantly (Tukey-Kramer test, *p* < 0.05); DAT: days after treatment.

As for colonization intensity (percentage of colonized leaf pieces), the presence of *B. bassiana* was detected in 64–85% of melon and 62–82% of strawberry leaf pieces (Figure 2).

As expected, the differences among various treatments in both plants proved to be statistically significant (melon: F = 16.475, df = 3, 350, *p* < 0.001 and strawberry: F = 15.845, df = 3, 350, *p* < 0.001). Wild strain AP0101 colonized significantly more leaf pieces than the other strains (Figure 2) in both plants. Three-way ANOVA tests revealed that plant species and fungal strain had a significant effect on colonization intensity, while the time interval from treatment (DAT) showed no significant influence (Table 1).

### 3.2. Insect Pest Population

**Figure 3:**
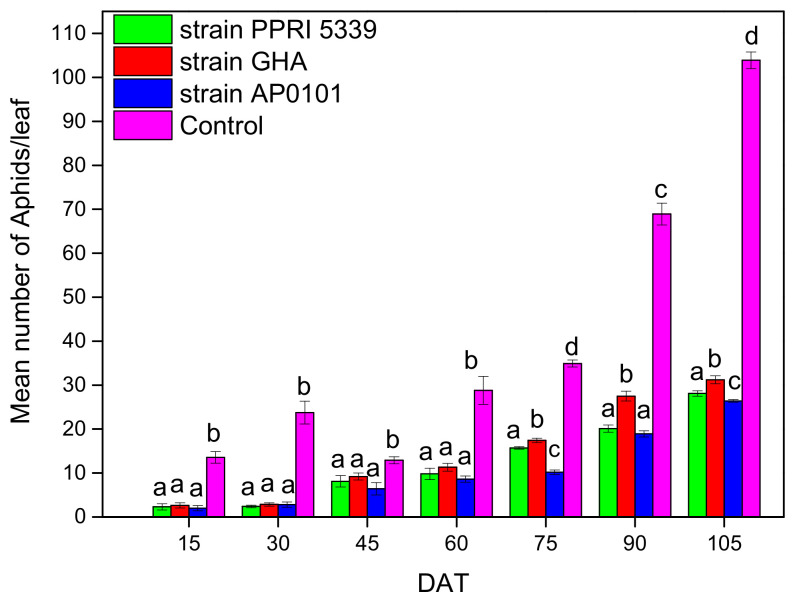
The average number of *A. gossypii* nymphs per leaf on *C. melo* plants inoculated with *B. bassiana*. Each treated plant was watered with 10 mL of 10^8^ conidia/mL suspension at the stage of four leaves (May 2022), 25 leaves were sampled and examined from each treatment plot, only 3rd–4th instar nymphs were counted, means of the same DAT marked with the same letter did not differ significantly (Tukey-Kramer test, *p* < 0.05); DAT: days after treatment.

The average population of *A. gossypii* nymphs per treatment in the melon crop was recorded. More specifically, in all seven samplings, the variation in the average number of *A. gossypii* nymphs was statistically significant among treatments (F = 14.771, df = 3, 511, *p* < 0.001). From the first sampling, the average number of aphids was significantly higher in the control than in the treatments (Figure 3). The average change in aphid population at the end of the experiment, after 105 days, was 28.1 ± 0.6 aphids for endophytic *B. bassiana* strain PPRI5339, 31.2 ± 0.9 aphids for GHA, 26.4 ± 0.3 aphids for AP0101, while in control plants, the aphid population was 103.9 ± 1.9 aphids (Figure 3). Fewer aphids were almost always counted on plants treated with wild strain AP0101 than on plants inoculated with other strains. These differences were significant in most cases (Figure 3).

**Figure 4:**
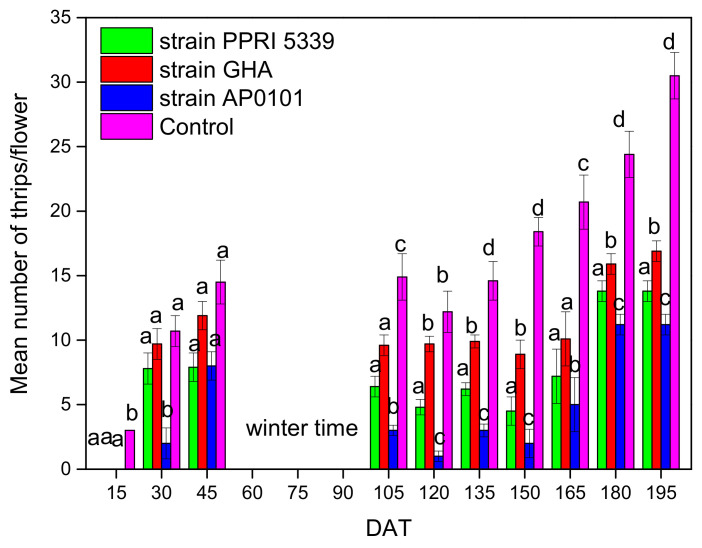
The average number of *F. occidentalis* adults per flower on *Fragaria* sp. plants inoculated with *B. bassiana*. Each treated plant was watered with 10 mL of 10^8^ conidia/mL suspension at the stage of four leaves (November 2021), and after 4 months (March 2022), 25 flowers were sampled and examined from each treatment plot; only adults were counted, means of the same DAT marked with the same letter did not differ significantly (Tukey-Kramer test, *p* < 0.05); DAT: days after treatment.

During the first months of strawberry growth, temperatures were not favorable for the insects, some individuals were recorded until early December, after which no insect population was detected until February (Figure 4). After overwintering, the average total number of *F. occidentalis* was significantly lower in control compared to the inoculated plants (F = 12.201, df = 3, 1011, *p* < 0.001). Similar to the case of *A. gossypii*, the plants inoculated with wild *B. bassiana* strain AP0101 recorded significantly lower thrip infestation compared to the commercial strains, especially after the winter period (Figure 4). The average change in *F. occidentalis* population at the end of the experiment per leaflet, after 210 days, was 13.9 ± 0.8 thrips for endophytic *B. bassiana* strain PPRI5339, 16.9 ± 0.8 thrip for GHA, 11.2 ± 0.8 thrips for AP0101 and 30.5 ± 1.8 thrips for the control (Figure 4).

**Figure 5:**
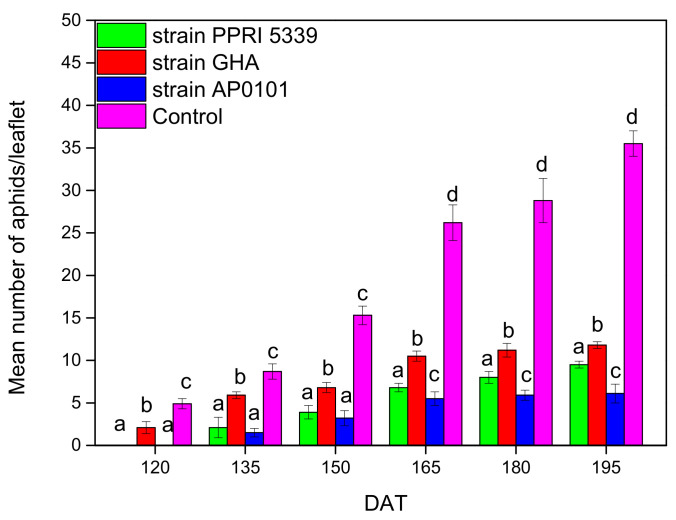
The average number of *C. fragaefolii* nymphs per leaflet on *Fragaria* sp. plants inoculated with *B. bassiana*. Each treated plant was watered with 10 mL of 10^8^ conidia/mL suspension at the stage of four leaves (November 2021), and after 4 months (March 2022), 25 leaves were sampled and examined from each treatment plot; only 3rd–4th instar nymphs were counted, means of the same DAT marked with the same letter did not differ significantly (Tukey-Kramer test, *p* < 0.05); DAT: days after treatment.

As for the monitoring of *C. fragaefolii*, the mean population size was significantly higher in the control (F = 15.371, df = 3, 511, *p* < 0.001), starting from the first sampling (April 2022). As for the average change in aphid population at the end of the experiment, there appeared to be statistically significant differences between treatments; after 90 days, there were 9.5 ± 0.4 aphids for the endophytic *B. bassiana* strain PPRI5339, 11.8 ± 0.4 aphids for GHA and 6.1 ± 1.1 aphids for AP0101, while the aphid population in the control plants was 35.5 ± 1.5 aphids (Figure 5). The wild-strain GHA again performed better than the commercial strains, but this time, the differences with strain PPRI5339 were not always significant (Figure 5).

### 3.3. Effect on Plant Growth

The evaluation of the morphological features of the tested plants was based on the recording of the number of leaves, flowers and fruits. In general, inoculation with *B. bassiana* AP0101 and *B. bassiana* PPRI 5339, resulted in better growth compared to *B. bassiana* GHA and the untreated plants. The former plants were more vigorous, greener and larger than the latter, which were more stressed.

**Figure 6:**
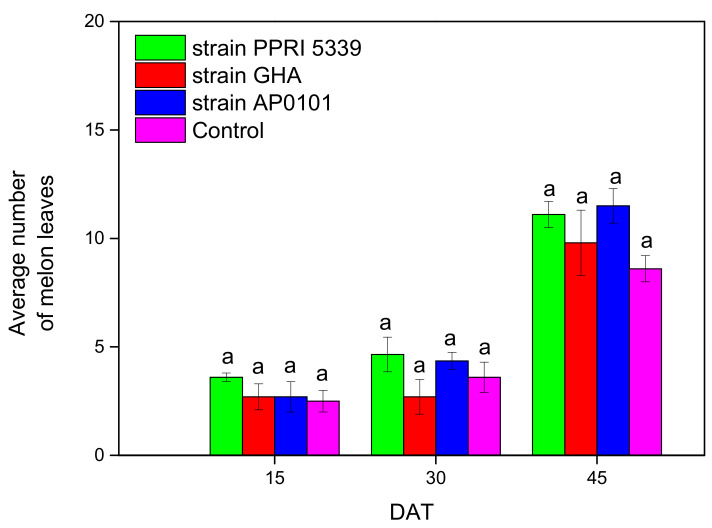
Average number of leaves per plant of *C. melo* inoculated with *B. bassiana.* Treated plants were watered with 10 mL of *B. bassiana* conidial suspension of 10^8^ conidia/mL at the stage of four leaves, means of the same DAT marked with the same letter did not differ significantly (Tukey-Kramer test, *p* < 0.05); DAT: days after treatment.

In melon plants, the number of leaves did not change significantly after 45 days (F = 3.112, df = 3, 511, *p* = 0.214) (Figure 6).

**Figure 7:**
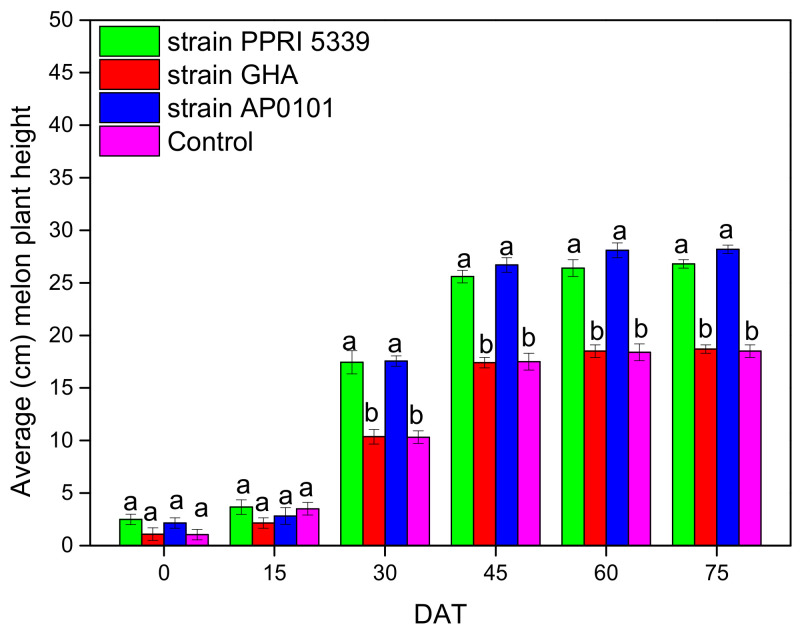
Average height (cm) (mean ± sd) of *C. melo* plants inoculated with *B. bassiana*. Treated plants were watered with 10 mL of *B. bassiana* conidial suspension of 10^8^ conidia/mL on the stage of four leaves, means of the same DAT marked with the same letter did not differ significantly (Tukey-Kramer test, *p* < 0.05); DAT: days after treatment.

Melon height was measured as 10.5 cm (Control) to 17.6 cm (*B. bassiana* AP0101) (Figure 7), and the differences were statistically significant (F = 10.365, df = 3, 511, *p* = 0.010).

**Figure 8:**
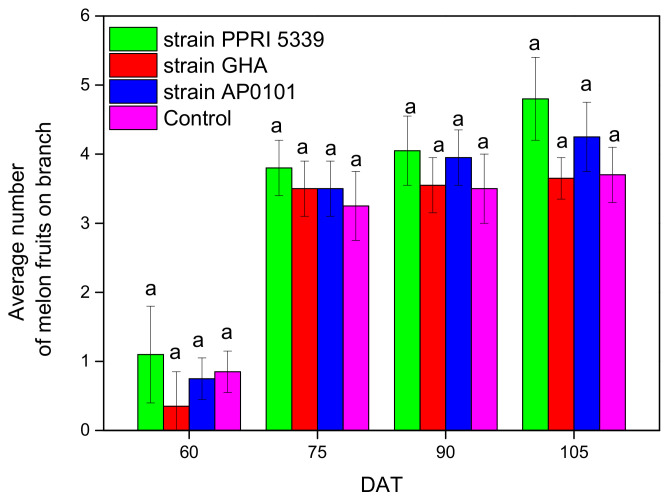
Average number of fruits per *C. melo* branch inoculated with *B. bassiana*. Treated plants were watered with 10 mL of *B. bassiana* conidial suspension of 10^8^ conidia/mL at the stage of four leaves, and means of the same DAT marked with the same letter did not differ significantly (Tukey-Kramer test, *p* < 0.05); DAT: days after treatment.

During harvest, plants inoculated with *B. bassiana* AP0101 and *B. bassiana* PPRI 5339 bore more fruit than the remaining plants. (Figure 8). The number of fruits per plant was counted from 3.6 ± 0.3 (*B. bassiana* GHA) to 4.8 ± 0.6 (*B. bassiana* PPRI 5339) after two months (Figure 8), and no statistically significant differences were found between fruits per plant (F = 5.365, df = 3, 123, *p* = 0.289).

**Figure 9:**
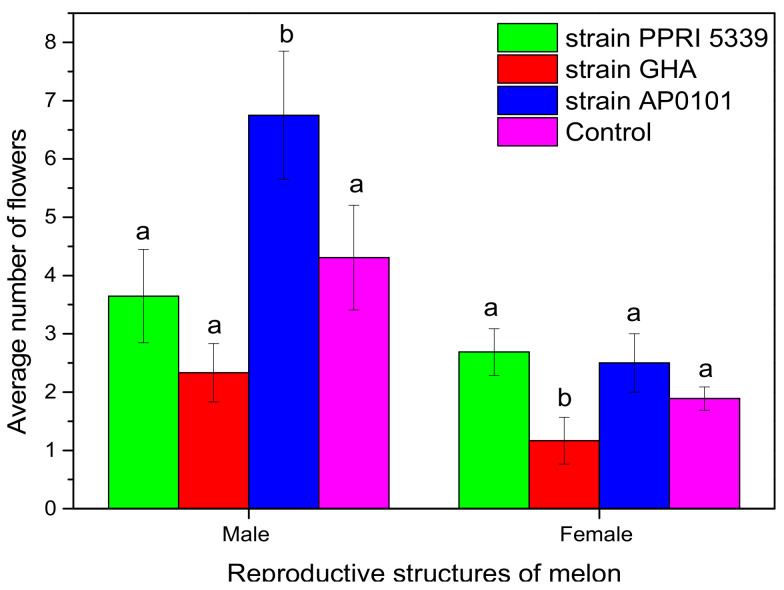
Average number of male and female flowers per *C. melo* plants inoculated with *B. bassiana.* Treated plants were watered with 10 mL of *B. bassiana* conidial suspension of 10^8^ conidia/mL on the stage of four leaves, and means of the same flower type marked with the same letter did not differ significantly (Tukey-Kramer test, *p* < 0.05).

The average number of male and female flowers, of *C. melo* was also recorded. The number of male flowers per plant ranged from 2.3 ± 0.5 (*B. bassiana* GHA) to 6.8 ± 1.1 (*B. bassiana* AP0101) and of female flowers from 1.1 ± 0.4 (*B. bassiana* GHA) to 2.7 ± 0.4 (*B. bassiana* PPRI 5339) (Figure 9). Statistically significant differences were recorded between male (F = 8.766, df = 3, 214, *p* = 0.038) and female flowers (F = 6.112, df = 3, 214, *p* = 0.048). The fewest flowers were counted in melon plants treated with *B. bassiana* GHA.

**Figure 10:**
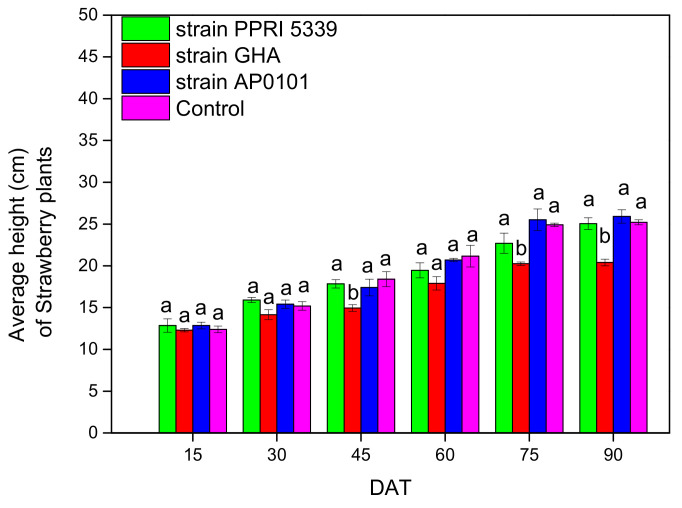
Average height (cm) of *Fragaria* sp. plants inoculated with *B. bassiana.* Each treated plant was watered with 10 mL of 10^8^ conidia/mL suspension at the stage of four leaves (November 2021), and after 4 months (March 2022), means of the same DAT marked with the same letter did not differ significantly (Tukey-Kramer test, *p* < 0.05); DAT: days after treatment.

The growth results of strawberry plants were quite similar to those of melon plants. Plant height after 90 days was measured between 20.4 ± 0.4 cm (*B. bassiana* GHA) and 25.9 ± 0.8 cm (*B. bassiana* AP0101), appearing to be statistically marginally different (F = 9.111, df = 3, 312, *p* = 0.042) (Figure 10).

**Figure 11:**
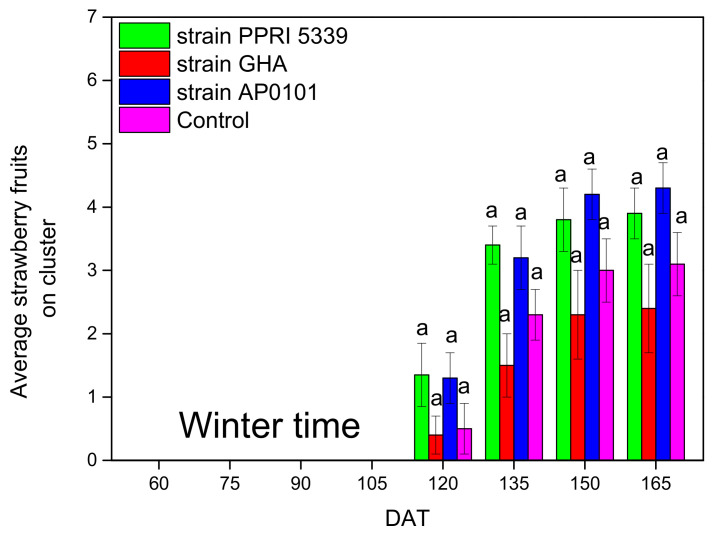
Average number of fruits per *Fragaria* sp. plants inoculated with *B. bassiana.* Each treated plant was watered with 10 mL of 10^8^ conidia/mL suspension at the stage of four leaves (November 2021), and after 4 months (March 2022), means of the same DAT marked with the same letter did not differ significantly (Tukey-Kramer test, *p* < 0.05); DAT: days after treatment.

The average number of fruits per plant after 120 days ranged from 2.4 ± 0.7(*B. bassiana* GHA) to 4.3 ± 0.4 (*B. bassiana* AP0101) with insignificant differences (F = 6.365, df = 3, 123, *p* = 0.131). The same pattern was observed in the following measurement points (Figure 11).

**Figure 12:**
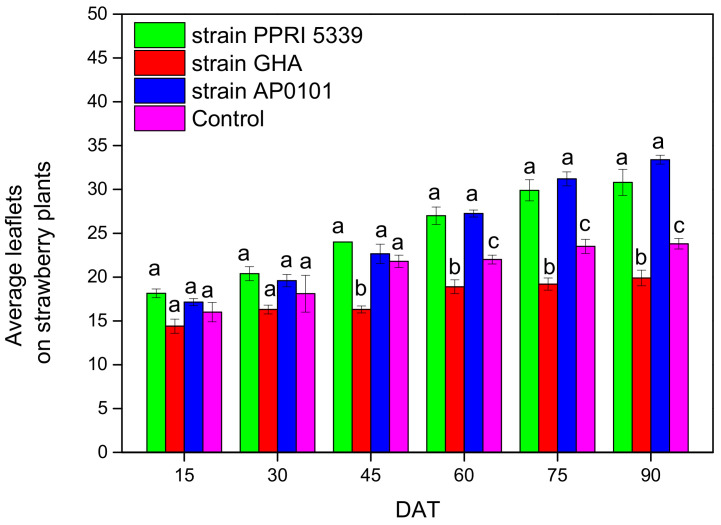
Average number of leaflets of *Fragaria* sp. plants inoculated with *B. bassiana.* Each treated plant was watered with 10 mL of 10^8^ conidia/mL suspension at the stage of four leaves (November 2021), and after 4 months (March 2022), means of the same DAT marked with the same letter did not differ significantly (Tukey-Kramer test, *p* < 0.05); DAT: days after treatment.

The number of leaflets of *Fragaria* spp. counted after 90 days ranged from 19.9 ± 0.9 (*B. bassiana* GHA) to 33.4 ± 0.5 *(B. bassiana* AP0101), with statistically significant differences (F = 14.365, df = 3, 123, *p* < 0.001) (Figure 12).

**Figure 13:**
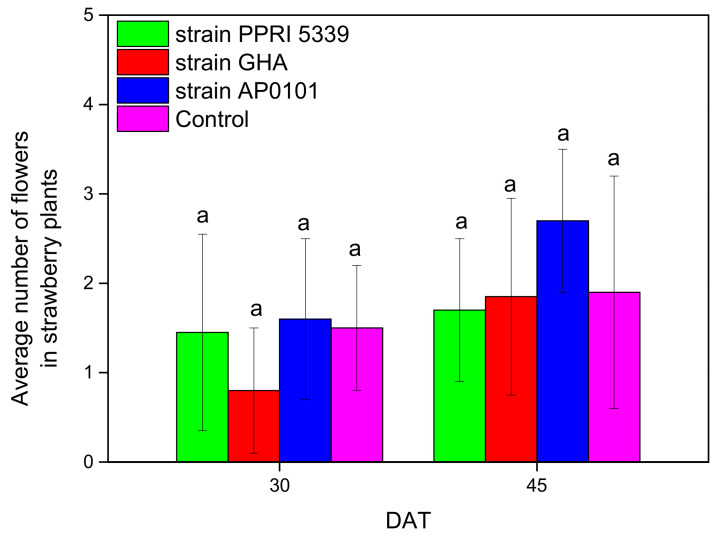
Average number of flowers per *Fragaria* sp. Plants inoculated with *B. bassiana.* Each treated plant was watered with 10 mL of 10^8^ conidia/mL suspension at the stage of four leaves (November 2021), and after 4 months (March 2022), means of the same DAT marked with the same letter did not differ significantly (Tukey-Kramer test, *p* < 0.05); DAT: days after treatment.

Finally, insignificant differences in the number of flowers were recorded among the experimental plants after 45 days (F = 6.766, df = 3, 123, *p* = 0.338) (Figure 13).

## 4. Discussion

The climatic conditions in Greece are optimal for the cultivation of melon and strawberry plants. Especially in the production of strawberries, Greece has become a globally competitive producer. However, most of the growing areas still rely on conventional methods for pest management and fertilization. The use of EPF, in addition to its numerous advantages over conventional insecticides, has a key role in sustainable crop pest management programs [24].

The EPF *B. bassiana* is of great interest in agriculture because it can colonize a range of plant species [25], it can effectively control not only pest infestations and also fungal diseases and it can trigger physiological mechanisms that promote nutrient uptake and plant growth and increase tolerance to abiotic stress and drought [17,18,26,27]. Numerous plants have been artificially inoculated with *B. bassiana* using various methods ([28] and refs therein) demonstrating its colonizing capability. This was also confirmed in our experiments, where all tested isolates colonized plant tissues very successfully, being detected on all sampled leaves of melon and strawberry as early as seven days after inoculation.

Previous field studies evaluated the effects of endophytic *B. bassiana* (strain GHA) in commercial strawberry plants [29,30,31,32] and reported a positive impact on plant growth and yield, which is consistent with our study. When strawberry plants were sprayed with *B. bassiana* (strain GHA) in the field, the colonization rate in leaves ranged from 18.8 to 43.8% [29]. This rate is much lower than ours (100%), which is due to the different inoculation methods, agronomic practices and crop variety. When greenhouse strawberries received a soil application of *B. bassiana,* they appeared to be healthier than untreated ones, although the number of leaves did not increase significantly [32]. In a recent field study, strawberry plants root-inoculated with *B. bassiana* showed >50% reduction in the population of *Tetranychus urticae* Koch (Acari: Tetranychidae) and 50% fewer leaves damaged by Coleoptera, while surprisingly, there was no effect on whitefly and thrip numbers [33]. Contradictory results are not uncommon in field studies with fungal endophytes, as their efficacy in pest control is highly dependent on the inoculation method, plant and pest species, EPF strain, farming practices and environmental conditions [28,34].

Only a few reports are available regarding the endophytic ability of *B. bassiana* on melon plants. Spraying with conidial suspension resulted in 100% colonization of leaves directly sprayed with the fungus, but only 40–60% of unsprayed leaves of the same plants [35,36]. The differences with our study on colonization rates on melon leaves are due to the different inoculation method (our conidial suspension was applied in soil). It has been well documented that the method of application drastically changes the level of colonization [28,37]. When another sucking pest, the whitefly *Bemisia tabaci* Gennadius (Hemiptera: Aleyrodidae), was fed on melon leaves colonized with *B. bassiana,* it suffered 66.3–87.9% mortality compared to untreated leaves [35]. Similar results were also recorded in chewing insects, the larvae of the cotton leafworm *Spodoptera littoralis* (Boisduval) (Lepidoptera: Noctuidae), when they were also fed on the same melon leaves [36].

Endophytic strains of *B. bassiana* have been described as highly virulent against aphids, in particular by reducing their population, fecundity and feeding behavior, such as *Sitobion avenae* F. [38], *Aphis gossypii* Glover [39,40,41], *Myzus persicae* Sulzer [42], *Aphis glycines* Matsumura [43], *Acyrthosiphon pisum* (Harris) [44] and *Aphis fabae* Scopoli (Hemiptera: Aphididae) [44]. Colonization by *B. bassiana* may not only increase mortality but also alter aphid feeding behavior as has been found with *M. persicae* on strawberries [45] and *A. gossypii* on melons [46], reducing virus transmission rates. A similar aphidicidal effect of the endophytic strains *B. bassiana* was observed in the present study on melon (69.9–74.5% reduction of *A. gossypii* population) and strawberry (66.7–82.8% reduction of *C. fragaefolii* population) plants.

To the best of our knowledge, this is the first field study to evaluate the efficacy of *B. bassiana* strains in controlling thrips when established inside plant tissues. In our study, the population decline of *F. occidentalis* on inoculated strawberry plants ranged from 44.5% to 63.2% on the last day of sampling. Several fungal endophytes have also been studied for thrips management, with promising results [47,48,49], while others were found to be ineffective [50,51].

During our observations of *C. melo*—*A. gossypii* and *Fragaria* sp.—*F. occidentalis* interactions, it was found that plants inoculated with wild *B. bassiana* strain AP0101 demonstrated the lowest pest population in both cases, followed by *B. bassiana* PPRI 5339. These results highlight the fact that these strains can undoubtedly become effective agricultural tools, as they exhibited high levels of endophytic activity, significantly promoted plant growth, increased yield in two completely different plant species and significantly contained two different populations of sucking pests. In the case of *B. bassiana* GHA, the results were also remarkable, as pest infestations were consistently lower than in the control in both *A. gossypii* and *F. occidentalis*, although the results were not comparable to those of the other two strains.

Sucking insects have a simple body structure, feeding and reproducing efficiently, while most of the nutrient uptake is invested in the production of nymphs [52]. Infection by endophytes is dependent on the genetic and environmental makeup of the insect population. The odor or taste of the host plant comes from nutrients and odd compounds that are converted into complex sensorial inputs in herbivorous insects [53]. These inputs are interpreted by the insect’s central nervous system to determine if a particular plant is a suitable host [54].

Plants inoculated with *B. bassiana* PPRI 5339 and the wild strain demonstrated richer growth (height, number of leaves) in both plant species compared to the control and the *B. bassiana* GHA treatment. In harvested fruits, EPF colonization generally improved crop yield, but differences among treatments were borderline statistically insignificant. Similar growth promotion in strawberry plants has been reported previously [33,34,35,36], whereas this study is the first to report enhancement of plant growth and production of melon plants by inoculation by *B. bassiana*. The positive effect of endophytic strains of *B. bassiana* on plant growth and yield has been well documented in several other crops, such as coffee [55], maize [56], tomato [57], soybean [58], barley [59], broad bean [60], grapevine [61], tobacco [62] and cotton [11].

Our results demonstrate the efficacy of *B. bassiana* in colonizing melon and strawberry plants while promoting plant growth and resistance against two major sucking pests that cause great damage to plants and transmit viruses. This report also highlights the fact that researchers and companies should not be satisfied with the current commercial products and continue research into new strains. Our results show that *B. bassiana* wild strain AP0101 displayed greater efficacy as an endophyte, as an entomopathogen and as a promoter of plant growth compared to commercial strains.

It is becoming apparent that the crop protection landscape will change radically over the next decade. This is reflected in particular in the decisions of the European Union under the “Green Deal” which aims to increase of organic farming from 8% to 25% and to reduce the use of chemical pesticides by 50% by 2030 [63]. Among all the alternatives to achieve the above goals, the use of endophytes is probably the most promising. Therefore, research should be intensified to discover new strains and evaluate them not only in the laboratory but also in the field, as in the present study.

## 5. Conclusions

In conclusion, our research reveals that treatments with endophytes in strawberries and melons induced systemic responses in the plant, that then affect aphid and thrip populations by disrupting reproduction or causing feeding disorders. However, more research is needed to discover the mechanisms by which these fungi regulate the number of sucking pests. Because this information has not been available, these studies are essential for evaluating the potential of non-chemical strategies for pest management in melon and strawberry plants. Plant treatments with endophytes reduced pest populations compared to controls, but this effect depended on the isolate; these isolates have a broad spectrum of action that can be utilized for the biological control of these pests. Microbial biopesticides, either separately or in combination with chemicals and other options, can provide effective, human-friendly and environmentally safe pest management.

## Figures and Tables

**Table 1:** ANOVA results for main effects and interactions for the intensity of *B. bassiana* colonization.

Source	df	F	*p*
Fungus Strain	3	25,365	<0.001
DAT	2	1530	0.207
Plant Species	1	12,312	<0.001
Fungus Strain × DAT	6	1420	0.135
Fungus Strain × Plant Species	3	8405	<0.001
DAT × Plant Species	2	1813	0.238
Fungus Strain × DAT × Plant Species	6	3069	<0.001
Error	636		
Total	804		
Corrected Total	803		

## Data Availability

Not applicable.

## References

[1] Jiang Y., Zhang C.X., Chen R., He S.Y. (2019). Challenging battles of plants with phloem-feeding insects and prokaryotic pathogens. Proc. Natl. Acad. Sci. USA.

[2] Reitz S.R. (2009). Biology and Ecology of the Western Flower Thrips (Thysanoptera: Thripidae): The Making of a Pest. Fla. Entomol..

[3] Villada E.S., Gonzalez E.G., Lopez-Sese A.I., Castiel A.F., Gomez-Guillamon M.L. (2009). Hypersensitive response to *Aphis gossypii* Glover in melon genotypes carrying the Vat gene. J. Exp. Bot..

[4] Cédola C., Greco N. (2010). Presence of the aphid, *Chaetosiphon fragaefolii*, on strawberry in Argentina. J. Insect Sci..

[5] He Z., Guo J., Reitz S.R., Lei Z., Wu S. (2020). A global invasion by the thrip, *Frankliniella occidentalis*: Current virus vector status and its management. Insect Sci..

[6] Lavandero B., Rojas P., Ramirez C.C., Salazar M., Caligari P.D. (2012). Genetic structure of the aphid, *Chaetosiphon fragaefolii*, and its role as a vector of the Strawberry Yellow Edge Virus to a native strawberry, *Fragaria chiloensis* in Chile. J. Insect Sci..

[7] Benatto A., Penteado S.C., Zawadneak M.A.C. (2019). Performance of *Chaetosiphon fragaefolii* (Hemiptera: Aphididae) in different strawberry cultivars. Neotrop. Entomol..

[8] Goettel M.S., Koike M., Kim J.J., Aiuchi D., Shinya R., Brodeur J. (2008). Potential of *Lecanicillium* spp. for management of insects, nematodes and plant diseases. J. Invertebr. Pathol..

[9] Ownley B.H., Gwinn K.D., Vega F.E. (2010). Endophytic fungal entomopathogens with activity against plant pathogens: Ecology and evolution. BioControl.

[10] Jaber L.R., Salem N.M. (2014). Endophytic colonization of squash by the fungal entomopathogen *Beauveria bassiana* (Ascomycota: Hypocreales) for managing Zucchini yellow mosaic virus in cucurbits. Biocontrol Sci. Technol..

[11] Lopez D.C., Sword G.A. (2015). The endophytic fungal entomopathogens *Beauveria bassiana* and *Purpureocillium lilacinum* enhance the growth of cultivated cotton (*Gossypium hirsutum*) and negatively affect survival of the cotton bollworm (*Helicoverpa zea*). Biol. Control.

[12] Bruck D.J. (2009). Fungal entomopathogens in the rhizosphere. The Ecology of Fungal Entomopathogens.

[13] Quesada-Moraga E., Herrero N., Zabalgogeazcoa Í. (2014). Entomopathogenic and nematophagous fungal endophytes. Advances in Endophytic Research.

[14] Tan R.X., Zou W.X. (2001). Endophytes: A rich source of functional metabolites. Nat. Prod. Rep..

[15] Strobel G.A. (2003). Endophytes as sources of bioactive products. Microbes Infect..

[16] Jaber L.R., Araj S.E. (2018). Interactions among endophytic fungal entomopathogens (Ascomycota: Hypocreales), the green peach aphid *Myzus persicae* Sulzer (Homoptera: Aphididae), and the aphid endoparasitoid *Aphidius colemani* Viereck (Hymenoptera: Braconidae). Biol. Control.

[17] Sinno M., Ranesi M., Di Lelio I., Iacomino G., Becchimanzi A., Barra E., Molisso D., Pennacchio F., Digilio M.C., Vitale S. (2021). Selection of endophytic Beauveria bassiana as a dual biocontrol agent of tomato pathogens and pests. Pathogens.

[18] Gupta R., Keppanan R., Leibman-Markus M., Rav-David D., Elad Y., Ment D., Bar M. (2022). The entomopathogenic fungi *Metarhizium brunneum* and *Beauveria bassiana* promote systemic immunity and confer resistance to a broad range of pests and pathogens in tomato. Phytopathology.

[19] Mantzoukas S., Pettas I., Lagogiannis I. (2020). Stored product pests as models for trapping entomopathogenic fungi from olive tree orchards in Western Greece. J. Stored Prod. Res..

[20] Mantzoukas S., Chondrogiannis C., Grammatikopoulos G. (2015). Effects of three endophytic entomopathogens on sweet sorghum and on the larvae of the stalk borer *Sesamia nonagrioides*. Entomol. Exp. Appl..

[21] González-Zamora J.E., Garcia-Marí F. (2013). The efficiency of several sampling methods for *Frankliniella occidentalis* (Thysanoptera: Thripidae) in strawberry flowers. J. Appl. Entomol..

[22] IBM Corp (2020). IBM SPSS Statistics for Windows.

[23] Barnett H.L., Hunter B.B. (1998). Illustrated Genera of Imperfect Fungi.

[24] Charnley A.K., Collins S.A., Kubicek C., Druzhinina I. (2007). Entomopathogenic fungi and their role in pest control. The Mycota IV. Environmental and Microbial Relationships.

[25] Mantzoukas S., Tamez-Guerra P., Zavala-Garcia F., Lagogiannis I., Ek-Ramos M.J. (2022). Entomopathogenic fungi tested in planta on pepper and in field on sorghum, to control commercially important species of aphids. World J. Microbiol. Biotechnol..

[26] Bamisile B.S., Dash C.K., Akutse K.S., Keppanan R., Wang L. (2018). Fungal endophytes: Beyond herbivore management. Front. Microbiol..

[27] Quesada Moraga E. (2020). Entomopathogenic fungi as endophytes: Their broader contribution to IPM and crop production. Biocontrol Sci. Technol..

[28] Mantzoukas S., Eliopoulos P.A. (2020). Endophytic entomopathogenic fungi: A valuable biological control tool against plant pests. Appl. Sci..

[29] Dara S.K., Dara S.R., Dara S.S. (2013). Endophytic colonization and pest management potential of *Beauveria bassiana* in strawberries. J. Berry Res..

[30] Dara S.K. (2013). Entomopathogenic fungus Beauveria bassiana promotes strawberry plant growth and health. UCANR eJ Strawb. Veg..

[31] Dara S.K. (2016). First field study evaluating the impact of the entomopathogenic fungus Beauveria bassiana on strawberry plant growth and yield. UCANR eJ Strawb. Veg..

[32] Dara S.K. (2016). Endophytic *Beauveria bassiana* negatively impacts green peach aphids on strawberries. UCCE eNewsl. Strawb. Veg..

[33] Canassa F., Esteca F.C., Moral R.A., Meyling N.V., Klingen I., Delalibera I. (2020). Root inoculation of strawberry with the entomopathogenic fungi *Metarhizium robertsii* and *Beauveria bassiana* reduces incidence of the twospotted spider mite and selected insect pests and plant diseases in the field. J. Pest Sci..

[34] Mantzoukas S., Kitsiou F., Natsiopoulos D., Eliopoulos P.A. (2022). Entomopathogenic Fungi: Interactions and Applications. Encyclopedia.

[35] Garrido-Jurado I., Resquín-Romero G., Amarilla S.P., Ríos-Moreno A., Carrasco L., Quesada-Moraga E. (2017). Transient endophytic colonization of melon plants by entomopathogenic fungi after foliar application for the control of *Bemisia tabaci* Gennadius (Hemiptera: Aleyrodidae). J. Pest Sci..

[36] Resquín-Romero G., Garrido-Jurado I., Delso C., Ríos-Moreno A., Quesada-Moraga E. (2016). Transient endophytic colonizations of plants improve the outcome of foliar applications of mycoinsecticides against chewing insects. J. Invertebr. Pathol..

[37] Bamisile B.S., Dash C.K., Akutse K.S., Keppanan R., Afolabi O.G., Hussain M., Qasim M., Wang L. (2018). Prospects of endophytic fungal entomopathogens as biocontrol and plant growth promoting agents: An insight on how artificial inoculation methods affect endophytic colonization of host plants. Microbiol. Res..

[38] Mahmood Z., Steenberg T., Mahmood K., Labouriau R., Kristensen M. (2019). Endophytic Beauveria bassiana in maize affects survival and fecundity of the aphid *Sitobion avenae*. Biol. Control.

[39] Homayoonzadeh M., Esmaeily M., Talebi K., Allahyarm H., Reitz S., Michaud J.P. (2022). Inoculation of cucumber plants with Beauveria bassiana enhances resistance to *Aphis gossypii* (Hemiptera: Aphididae) and increases aphid susceptibility to pirimicarb. Eur. J. Entomol..

[40] Gurulingappa P., McGee P.A., Sword G. (2011). Endophytic *Lecanicillium lecanii* and *Beauveria bassiana* reduce the survival and fecundity of *Aphis gossypii* following contact with conidia and secondary metabolites. Crop Prot..

[41] Gurulingappa P., Sword G.A., Murdoch G., McGee P.A. (2010). Colonization of crop plants by fungal entomopathogens and their effects on two insect pests when in planta. Biol. Control.

[42] Mantzoukas S., Lagogiannis I. (2019). Endophytic colonization of pepper (*Capsicum annum*) controls aphids (*Myzus persicae* Sulzer). Appl. Sci..

[43] Clifton E.H., Jaronski S.T., Coates B.S., Hodgson E.W., Gassmann A.J. (2018). Effects of endophytic entomopathogenic fungi on soybean aphid and identification of *Metarhizium* isolates from agricultural fields. PLoS ONE.

[44] Akello J., Sikora R. (2012). Systemic acropedal influence of endophyte seed treatment on *Acyrthosiphon pisum* and *Aphis fabae* offspring development and reproductive fitness. Biol. Control.

[45] Manoussopoulos Y., Mantzoukas S., Lagogiannis I., Goudoudaki S., Kambouris M. (2019). Effects of three strawberry entomopathogenic fungi on the prefeeding behavior of the aphid *Myzus persicae*. J. Insect Behav..

[46] González-Mas N., Quesada-Moraga E., Plaza M., Fereres A., Moreno A. (2019). Changes in feeding behaviour are not related to the reduction in the transmission rate of plant viruses by *Aphis gossypii* (Homoptera: Aphididae) to melon plants colonized by *Beauveria bassiana* (Ascomycota: Hypocreales). Biol. Control.

[47] Muvea A.M., Meyhöfer R., Subramanian S., Poehling H.M., Ekesi S., Maniania N.K. (2014). Colonization of onions by endophytic fungi and their impacts on the biology of *Thrips tabaci*. PLoS ONE.

[48] Kiarie S., Nyasani J.O., Gohole L.S., Maniania N.K., Subramanian S. (2020). Impact of fungal endophyte colonization of maize (*Zea mays* L.) on induced resistance to thrips-and aphid-transmitted viruses. Plants.

[49] Muvea A.M., Subramanian S., Maniania N.K., Poehling H.M., Ekesi S., Meyhöfer R. (2018). Endophytic colonization of onions induces resistance against viruliferous thrips and virus replication. Front. Plant Sci..

[50] Demirozer O., Tyler-Julian K., Fundeburk J. (2004). Association of pep-per with arbusucular mycorrhizal fungi influence populations of the herbivore *Frankliniella occidentalis* (Thysanoptera: Thripidae). J. Entomol. Sci..

[51] Koschier E.H., Khaosaad T., Vierheilig H. (2007). Root colonization by the arbuscular mycorrhizal fungus *Glomus mosseae* and enhanced phosphorous levels in cucumber do not affect host acceptance and development of *Frankliniella occidentalis*. J. Plant Interact..

[52] Ramirez-Rodriguez D., Sánchez-Peña S.R. (2016). Endophytic *Beauveria bassiana* in *Zea mays*: Pathogenicity against Larvae of Fall Armyworm, *Spodoptera frugiperda*. Southwest. Entomol..

[53] Kuldau G.R., Bacon C. (2008). Clavicipitaceous endophytes: Their ability to enhance resistance of grasses to multiple stresses. Biol. Control.

[54] Eastop V.F., Harris K.F., Maramorosch K. (1977). Worldwide importance of aphids as virus vectors. Aphids as Virus Vectors.

[55] Posada F., Vega F.E. (2006). Inoculation and colonization of coffee seedlings (*Coffea arabica* L.) with the fungal entomopathogen *Beauveria bassiana* (Ascomycota: Hypocreales). Mycoscience.

[56] Russo M.L., Scorsetti A.C., Vianna M.F., Cabello M., Ferreri N., Pelizza S. (2019). Endophytic effects of *Beauveria bassiana* on corn (*Zea mays*) and its herbivore, *Rachiplusia nu* (Lepidoptera: Noctuidae). Insects.

[57] Prabhukarthikeyan R., Saravanakumar D., Raguchander T. (2014). Combination of endophytic *Bacillus* and *Beauveria* for the management of Fusarium wilt and fruit borer in tomato. Pest Manag. Sci..

[58] Russo M.L., Pelizza S.A., Vianna M.F., Allegrucci N., Cabello M.N., Toledo A.V., Mourellos C., Scorsetti A.C. (2019). Effect of endophytic entomopathogenic fungi on soybean *Glycine max* (L.) Merr. growth and yield. J. King Saud Univ. Sci..

[59] Veloz-Badillo G.M., Riveros-Ramírez J., Angel-Cuapio A., Arce-Cervantes O., Flores-Chávez B., Espitia-López J., Loera O., Garza-López P.M. (2019). The endophytic capacity of the entomopathogenic fungus *Beauveria bassiana* caused inherent physiological response in two barley (*Hordeum vulgare*) varieties. 3 Biotech.

[60] Jaber L.R., Enkerli J. (2016). Effect of seed treatment duration on growth and colonization of *Vicia faba* by endophytic *Beauveria bassiana* and *Metarhizium brunneum*. Biol. Control.

[61] Mantzoukas S., Lagogiannis I., Mpousia D., Ntoukas A., Karmakolia K., Eliopoulos P.A., Poulas K. (2021). *Beauveria bassiana* Endophytic Strain as Plant Growth Promoter: The Case of the Grape Vine *Vitis vinifera*. J. Fungi.

[62] Qin X., Zhao X., Huang S., Deng J., Li X., Luo Z., Zhang Y. (2021). Pest management via endophytic colonization of tobacco seedlings by the insect fungal pathogen *Beauveria bassiana*. Pest Manag. Sci..

[63] Fetting C. The European Green Deal. https://www.esdn.eu/fileadmin/ESDN_Reports/ESDN_Report_2_2020.pdf.

